# Sarcopenia increases the risk of early biliary infection after percutaneous transhepatic biliary stent placement

**DOI:** 10.3389/fonc.2022.1039987

**Published:** 2022-12-09

**Authors:** Qi Chen, Xun Lu, Zhong-kai Wang, Cheng Feng, Xi-Juan Yao, Jin-He Guo

**Affiliations:** ^1^ Center of Interventional Radiology and Vascular Surgery, Department of Radiology, Zhongda Hospital, Medical School, Southeast University, Nanjing, China; ^2^ Department of Urology, Affiliated Zhongda hospital of Southeast University, Nanjing, China; ^3^ Surgical Research Center, Institute of Urology, School of Medicine, Southeast University, Nanjing, China

**Keywords:** sarcopenia, biliary tract cancer, percutaneous transhepatic biliary stent, early biliary infection, microbiology

## Abstract

**Purpose:**

To assess the association between sarcopenia and the risk of early biliary infection (EBI) after percutaneous transhepatic biliary stent (PTBS) placement in patients with inoperable biliary tract cancer (BTC).

**Patients and methods:**

In this single center, retrospective observational study, patients diagnosed with inoperable BTC undergoing PTBS placement between January 2013 and July 2021 were enrolled. Preoperative sarcopenia was defined based on skeletal muscle mass measured by computed tomography images on the level of third lumbar vertebra within one month before PTBS placement. Patients were divided into two groups in accordance with the status of sarcopenia. Univariate and further multivariate logistic analyses were performed to determine predictors for EBI. Stratified and interactive analyses were conducted to investigate the stability of results. Further receiver operating characteristic curve was performed to determine the predictive value of sarcopenia on EBI after PTBS placement.

**Results:**

Totally, 134 patients were included in this retrospective study, with 45 (33.6%) patients characterized as sarcopenia. The incidence rate of EBI was 26.9% (36/134). Multivariate analyses demonstrated that sarcopenia [Odds ratio (OR), 2.75; 95%CI: 1.11–6.77; *P*=0.028], obstruction length (OR, 1.04; 95%CI: 1.00–1.08; *P*=0.030) and diabetes (OR, 2.46; 95%CI: 1.01–5.96; *P*=0.047) were significant predictors of EBI. There were no significant interactions in different subgroups (*P* for interaction > 0.05). Moreover, the areas under the curves (AUC) revealed that the combined index containing sarcopenia, obstruction length, and diabetes showed the better predictive value (AUC= 0.723) than either one alone.

**Conclusion:**

Sarcopenia increased the risk of EBI in patients with inoperable BTC after PTBS placement. Preoperative assessment of sarcopenia may aid in risk stratification. Patients with sarcopenia should be given intensive monitoring.

## Introduction

Biliary tract cancers (BTC) are the second most common hepatobiliary malignancies, including cholangiocarcinoma and gallbladder carcinoma (1, 2). Complete surgical resection is the cornerstone of curative therapy; however, most patients with BTC are initially diagnosed with unresectable or metastatic disease and presented with obstructive jaundice (3). For unresectable BTC, percutaneous transhepatic biliary stent (PTBS) placement is a palliative treatment to effectively alleviate jaundice (4).

Early biliary infection (EBI) includes cholangitis, liver abscess, cholecystitis and other biliary system related infection within 30 days after biliary intervention, which is a common and serious complication after PTBS placement (5, 6). According to the Tokyo Guideline, even with positive treatment, the reported mortality rate of acute cholangitis has stand in stark to 10% (7, 8). Thus, it is essential to explore potential risk factors of EBI for better preoperative care.

Sarcopenia is acknowledged as a progressive and chronic disease, which represents with lowered skeletal muscle mass (SMM) and decreased muscle function (9, 10). By evaluating the computed tomography (CT) images on the level of third lumbar vertebra (L3) slices, sarcopenia can act as a predictor for adverse outcomes in many tumor patients. Our previous study also found that sarcopenia could negatively predict survival in BTC patients after PTBS placement (11). Moreover, recent studies have pointed that sarcopenia was associated with postoperative infectious complications, such as colorectal cancer resection surgery (12), pancreaticoduodenectomy (13), and gastrectomy (14).

Previous studies have reported many risk factors of EBI after PTBS placement, such as diabetes, length and location of obstruction, history of biliary tract endoscopic intervention and the use of proton pump inhibitors (15, 16). However, till now, few studies have evaluated the association between sarcopenia and EBI after PTBS placement.

Therefore, we conducted a retrospective study to report the incidence rate of EBI and identify risk factors of EBI after PTBS placement. Furthermore, sarcopenia was also evaluated for its predictive value of EBI after PTBS placement.

## Patients and methods

### Study design

The study was a single institutional retrospective study. Patients diagnosed with unresectable BTC and underwent PTBS placement between January 2013 and July 2021 at authors’ hospital were included in the study. The criteria for inclusion contained: a) age older than 18 years; b) clinically or pathologically confirmed as unresectable BTC; c) biliary stent was performed for the first time, and prior percutaneous transhepatic biliary drainage (PTBD) was permitted. The exclusion criteria included: a) occurrence of biliary tract infection before PTBS placement; b) any surgery was performed within 30 days before PTBS; c) patients with previous immunodeficiencies or under immune suppressors; d) no EBI related death within 30 days; e) incomplete data or lost in follow-up.

The Ethics Committee of authors’ hospital approved this study. The requirement for informed consent was waived due to its retrospective nature.

### Data collection

The hospital information system (HIS) was screened and patients’ baseline characteristics were reviewed, including age, gender, Eastern Cooperative Oncology Group (ECOG) performance status, body mass index (BMI), Child-Pugh score, concomitant diabetes and gallstones, history of pre-procedural PTBD, and previous history of surgical or endoscopic intervention. Tumor related parameters such as etiology of tumor, obstruction site, obstruction length and stent position were collected. Preoperative laboratory examination results of white blood cell, serum level of albumin and total bilirubin and blood glucose were also included.

### PTBS procedure

All patients included in the study were conducted standard PTBS placement by an experienced interventional radiologist. The stent system is composed by two part, including an inner uncovered self-expandable metallic stent and an outer stent (Nanjing Micro-Tech Co. Ltd., Nanjing, China) carrying ^125^I seeds (CIAE-6711; Chinese Atomic Energy Science Institution, Beijing, China), which were pre-loaded into the surface of the outer one. As illustrated in our previous study (17), numbers, prescription dose and distribution of ^125^I seeds were programmed and calculated on the basis of Treatment Planning System (TPS, FTT Technology, Beijing, China).

**Figure 1:**
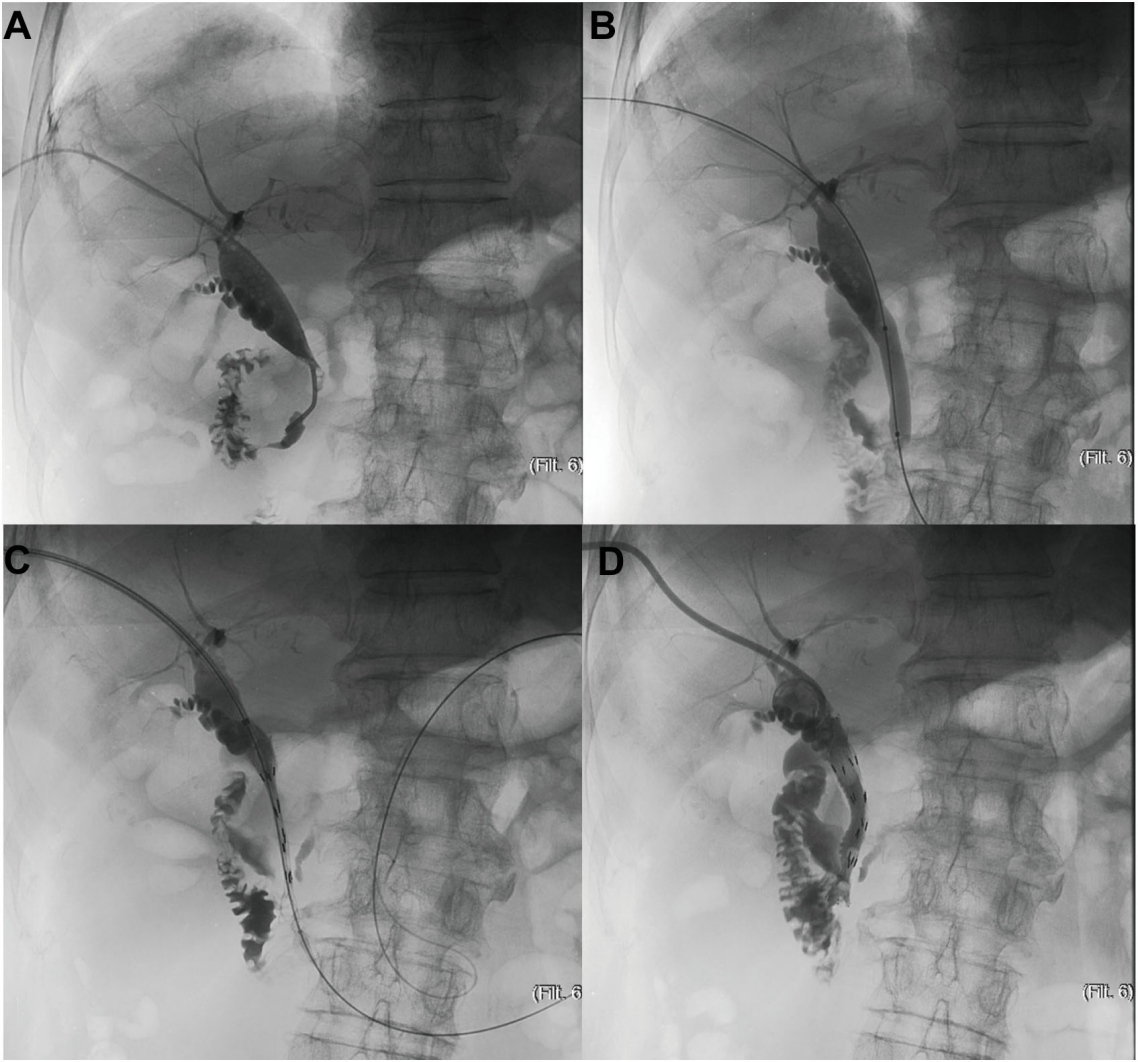
The schematic diagram of PTBS placement. **(A)** Confirmation of location and length of obstruction *via* cholangiography catheter **(B)** Dilation of passage with a balloon dilator catheter **(C)** Delivery of the outer ^125^I seed-loaded stent into the targeted location of lesion **(D)** Deployment of the inner stent to cover the outer one.

A channel was established under guidance of ultrasound or fluoroscopy, which already existed in patients with previous PTBD, for cholangiogram and further stent placement. The location and length of obstruction was confirmed *via* cholangiography catheter (**Figure 1A**), which was followed by dilating passage with a balloon dilator catheter (**Figure 1B**), then the outer ^125^I seed-loaded stent was successfully delivered into the targeted location of lesion (**Figure 1C**), and then immediately the inner one was deployed to cover the outer one (**Figure 1D**). The stent was placed according to the tumor location. When the tumor located at the lower 2 cm of the common bile duct, the stent was placed across the main duodenal papilla; Otherwise, the stent was placed above the main duodenal papilla. Finally, cholangiogram was utilized to confirm its patency.

Prophylactic antibiotics administration was conducted within 1 hour before PTBS placement. All patients were followed up for 1 month after PTBS placement.

### Definition and outcomes

EBI refers to biliary infection within 30 days after PTBS placement in the study. According to the Tokyo guidelines, the definition of EBI contains systemic inflammation (fever>38℃ or abnormal white blood cell counts or evaluated serum C-reactive protein levels), local signs of inflammation (exacerbation of pain in right upper quadrant or jaundice), exclusion of other infectious disease, combined with imaging evidence of biliary infection or positive bile or blood culture (7, 8). High-level obstruction is located on or above the common hepatic duct, including perihilar or intrahepatic obstruction. While the low-level one is situated below the cystic duct, which invades the common bile duct (18).

Sarcopenia was determined based on SMM by CT images in venous phase of the L3 slices within one month before PTBS placement, which included the psoas, paraspinal muscle, internal oblique, external oblique, rectus abdominis, and transverse abdominal. SMM was obtained by summing the pixel counts and multiplying by the per pixel surface area using the Magic Wand tool in Adobe Photoshop software by one well-trained radiologist, who was blinded to patients’ condition; and then SMM was calibrated by square of height to generate skeletal muscle index (SMI). Gender-specific thresholds were measured by calibrated SMI, and the index lower than the lowest tertiles was defined as sarcopenia.

The primary outcomes in the study were incidence and risk factors of EBI after PTBS placement in BTC patients. The secondary outcome was predictive value of sarcopenia on EBI after PTBS placement.

### Statistical analysis

For continuous variables, they were presented as mean with standard deviation (SD) or medians with interquartile range (IQR) according to the data distribution, while categorical variables were shown as frequency and percentages. Parameters between the groups were compared using a two-sample independent t-test or Mann–Whitney test for numerical data, and Pearson Chi-Square or Fisher’s exact tests for categorical data, as appropriate. Univariate and multivariate logistic analyses were conducted to evaluate the significance of variables for predicting EBI. For variables with *P*-value less than 0.05 in univariate analysis were further incorporated into multivariate logistic regression analysis. Interactions among subgroups were determined according to a multiplicative model to investigate whether the effect of sarcopenia on EBI was modified by other variables. The receiver operating characteristic (ROC) curve was constructed to assess the predictive value of variables for EBI by MedCalc program (version 20.015). Statistical analysis was conducted by the software Stata 15.1 (StataCorp, College Station, TX, USA). Finally, *P*-value < 0.05 was considered statistically significant in the study.

## Results

### Patients’ characteristics and clinical outcomes

**Figure 2:**
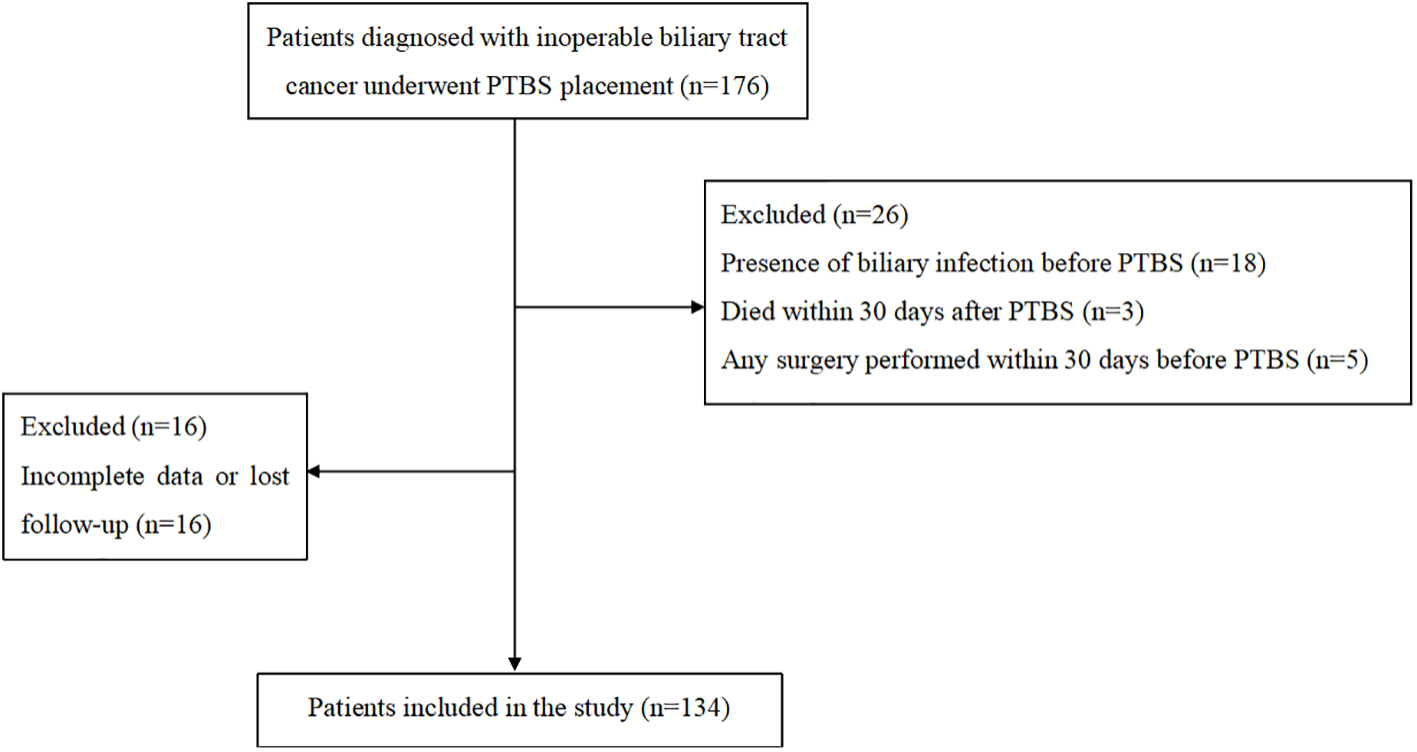
The flow chart of protocol in the study.

The flow chart was shown in **Figure 2**. Totally, 134 patients were included in the study. There were 66 male and 68 female patients with median age of 71 (IQR, 61-79) years. Overall, 45 patients were diagnosed with sarcopenia. Most patients have good physical quality as 82 (61.2%) patients obtained ECOG 0-1. For tumor related parameters, 101 (75.4%) patients were high-level obstruction and 33 (24.6%) patients were low-level obstruction with the median obstruction length to be 39 (IQR, 30-43) mm. The detailed results were shown in **Table 1**.

**Table 1:** Baseline characteristics of included patients.

Variable	Result
Total, n	134
Age, years, median (IQR)	71 (61-79)
Gender, n (%)	
Female	68 (50.8%)
Male	66 (49.3%)
ECOG, n (%)	
0-1	82 (61.2%)
2-3	52 (38.8%)
BMI, kg/m^2^, n (%)	
< 25	122 (91.0%)
≥ 25	12 (9.0%)
Child-Pugh Score, n (%)	
A	54 (40.3%)
B	80 (59.7%)
Etiology, n (%)	
Cholangiocarcinoma	102 (76.1%)
Gallbladder carcinoma	27 (20.2%)
AOV carcinoma	5 (3.7%)
Obstruction site, n (%)	
Low	33 (24.6%)
High	101 (75.4%)
Obstruction length, mm, median (IQR)	39 (30-43)
Diabetes, n (%)	
No	99 (73.9%)
Yes	35 (26.1%)
Gallstones, n (%)	
No	100 (74.6%)
Yes	34 (25.4%)
Pre-procedural PTBD, n (%)	
No	21 (15.7%)
Yes	113 (84.3%)
Previous surgical or endoscopic intervention, n (%)	
No	107 (79.8%)
Yes	27 (20.2%)
Stent position	
Suprapapillary	116 (86.6%)
Transpapillary	18 (13.4%)
Sarcopenia, n (%)	
No	89 (66.4%)
Yes	45 (33.6%)
WBC, x10^9^/L, median (IQR)	6.7 (5.0-8.4)
TB, umol/L, median (IQR)	96.4 (52.5-183.5)
ALB, g/L, median (IQR)	34.2 (31.9-37.7)
GLU, mmol/L, median (IQR)	5.3 (4.7-6.4)

ECOG, Eastern Cooperative Oncology Group; BMI, body mass index; AOV, ampulla of Vater; PTBD, percutaneous transhepatic biliary drainage; WBC, white blood cell; TB, total bilirubin; ALB, albumin; GLU, blood glucose.

**Figure 3:**
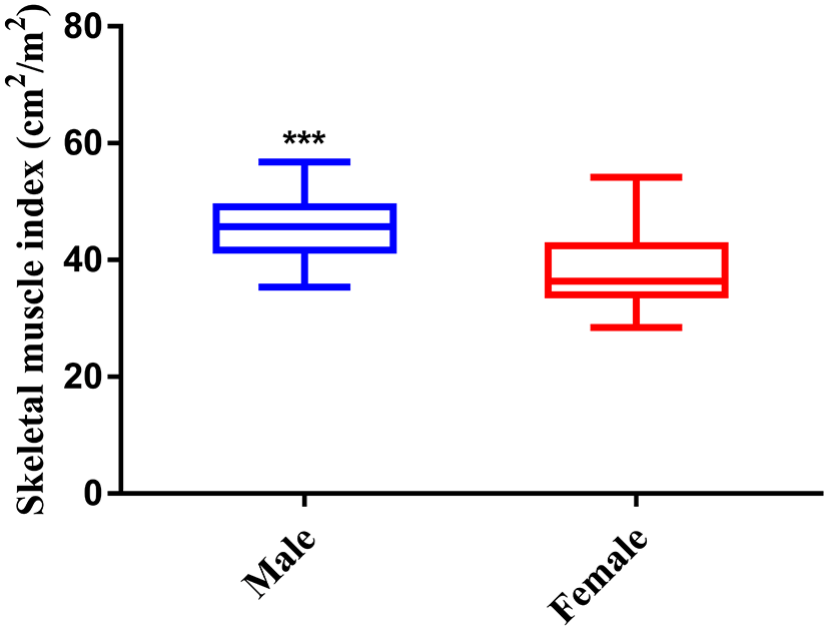
Skeletal muscle index between male and female patients. *** P < 0.001.

As shown in **Figure 3**, the index of SMI was significant higher in male than female, therefore, the gender-specific thresholds were determined, with the index lower than the lowest tertile one defined as sarcopenia. Accordingly, 45 (33.6%) patients were defined as sarcopenia in the study.

The incidence of EBI in the study was 26.9%, including cholangitis in 31 (86%) patients, cholecystitis in 2 (6%) patients, and liver abscess in 3 (8%) patients, respectively. Bile culture results were also shown in **Supplementary Table 1**.

### Risk factors associated with EBI

Univariate analysis was conducted to select the factors associated with EBI. The results revealed that ECOG (OR=2.58, 95%CI: 1.18-5.63, *P*=0.020), obstruction length (OR=1.05, 95%CI: 1.01-1.09, *P*=0.011), diabetes (OR=2.79, 95%CI: 1.22-6.36, *P*=0.015), and sarcopenia (OR=3.65, 95%CI: 1.64-8.12, *P*=0.001) were associated with EBI after PTBS placement (**Table 2**). Then these identified factors were further entered into multivariate analysis. Multivariate analysis results demonstrated that obstruction length (OR=1.04, 95%CI: 1.00-1.08, P=0.030), diabetes (OR=2.46, 95%CI: 1.02-5.96, P=0.047), and sarcopenia (OR=2.75, 95%CI: 1.11-6.77, P=0.028) remained to be independent risk factors of EBI after PTBS placement (**Table 3**).

**Table 2:** Univariate analysis of risk factors for EBI in patients after PTBS.

Variable	OR	95% CI	P
Gender			
Female	1.00	–	–
Male	1.93	0.89-4.21	0.099
Age	0.99	0.97-1.00	0.886
ECOG			
0-1	1.00	–	–
2-3	2.58	1.18-5.63	**0.020**
Child-Pugh Score			
A	1.00	–	**-**
B	1.78	0.79-4.01	0.166
Etiology			
Cholangiocarcinoma	1.00	–	–
Gallbladder carcinoma	0.72	0.26-1.96	0.520
AOV carcinoma	0.63	0.07-5.87	0.684
Obstruction site			
Low	1.00	–	–
High	1.50	0.59-3.83	0.401
Obstruction length	1.05	1.01-1.09	**0.011**
Diabetes			
Absence	1.00	–	–
Presence	2.79	1.22-6.36	**0.015**
Gallstones			
Absence	1.00		
Presence	1.44	0.61-3.35	0.405
Pre-procedural PTBD			
Absence	1.00		
Presence	1.21	0.41-3.58	0.731
BMI			
< 25 kg/m^2^	1.00	–	–
≥ 25 kg/m^2^	0.90	0.23-3.53	0.879
Previous surgical or endoscopic intervention			
Absence	1.00	–	–
Presence	0.56	0.19-1.60	0.278
Stent position			
Suprapapillary	1.00	–	–
Transpapillary	0.50	0.14-1.85	0.302
Sarcopenia			
Absence	1.00	–	–
Presence	3.65	1.64-8.12	**0.001**
WBC	1.09	0.94-1.26	0.250
TB	1.00	0.99-1.01	0.505
ALB	0.96	0.89-1.03	0.272
GLU	0.86	0.67-1.12	0.273

EBI, early biliary infection; PTBS, percutaneous transhepatic biliary stent; ECOG, Eastern Cooperative Oncology Group; AOV, ampulla of Vater; PTBD, percutaneous transhepatic biliary drainage; BMI, body mass index; WBC, white blood cell; TB, total bilirubin; ALB, albumin; GLU, blood glucose; CI, confidence interval; OR, odds ratio. The bold means P < 0.05.

**Table 3:** Multivariate analysis of risk factors for EBI in patients after PTBS.

Variables	OR	95%Cl	P
ECOG			
0-1	1.00	–	–
2-3	1.58	0.65-3.89	0.316
Obstruction length	1.04	1.00-1.08	**0.030**
Diabetes			
Absence	1.00	–	–
Presence	2.46	1.01-5.96	**0.047**
Sarcopenia			
Absence	1.00		
Presence	2.75	1.11-6.77	**0.028**

The bold means P < 0.05.

### Sarcopenia and EBI

Comparison of variables between two groups with or without sarcopenia were listed in **Supplementary Table 2**. Parameters including gender, age, BMI, and etiology showed no difference in patients with and without sarcopenia in the present study. Sarcopenic individuals were more likely to experience EBI than non-sarcopenic ones. Moreover, the results indicated that sarcopenia was associated with higher ECOG (*P*<0.001) and Child-Pugh score (*P*=0.002). Accordingly, the subgroup analysis was conducted to further investigate whether the effect of sarcopenia on EBI was affected by different variables.

**Figure 4:**
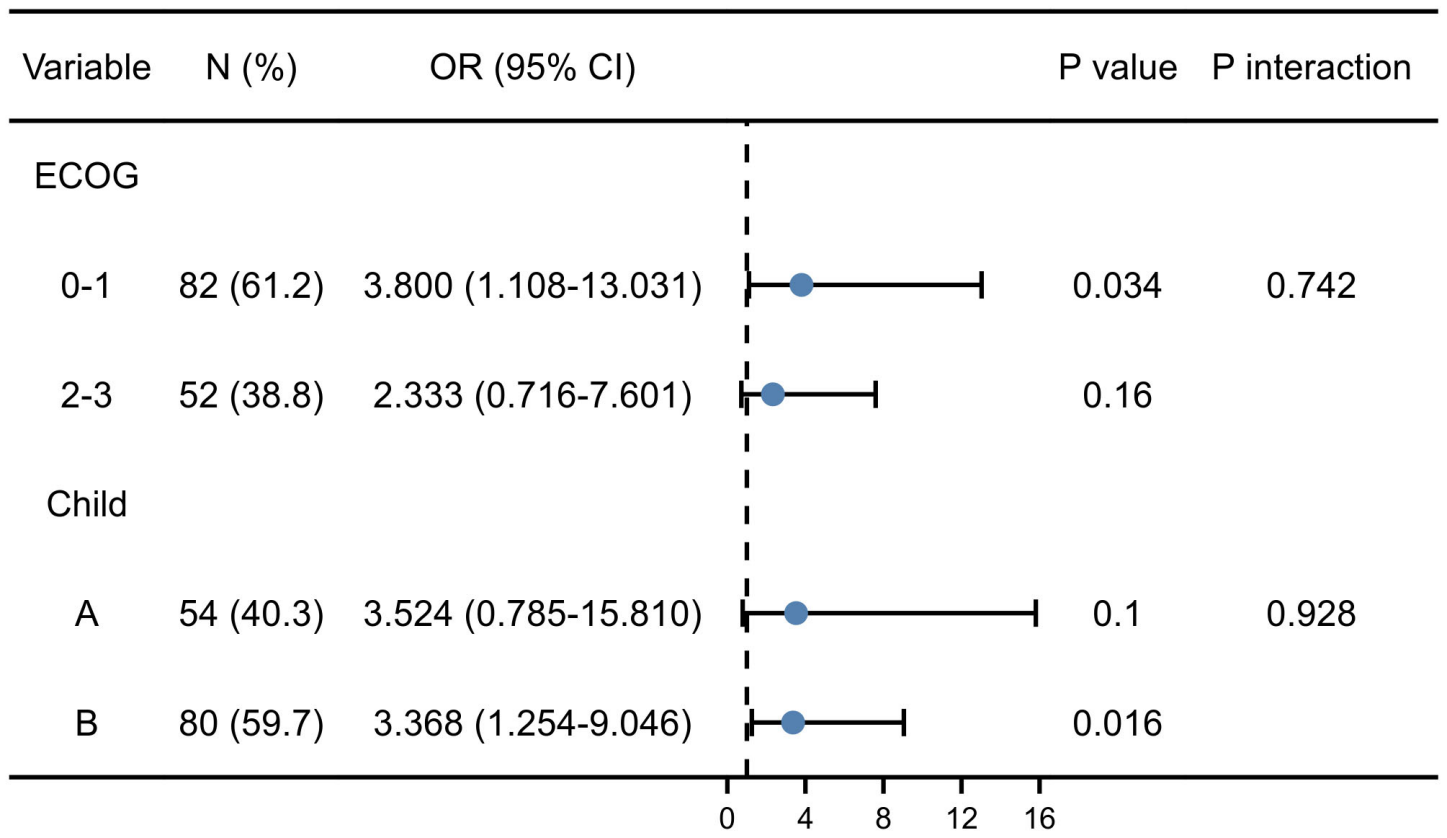
The forest plot of sarcopenia among ECOG and Child-Pugh score subgroups.

As shown in **Figure 4**, regardless of stratification by ECOG or Child-Pugh score, the predictive value of sarcopenia on EBI was consistent between different subgroups. Furthermore, the interaction analysis showed that the effect of sarcopenia on EBI was not affected by ECOG (*P*=0.742) and Child-Pugh score (*P*=0.928). Thus, the results further confirmed independent predictive value of sarcopenia on the risk of EBI.

**Figure 5:**
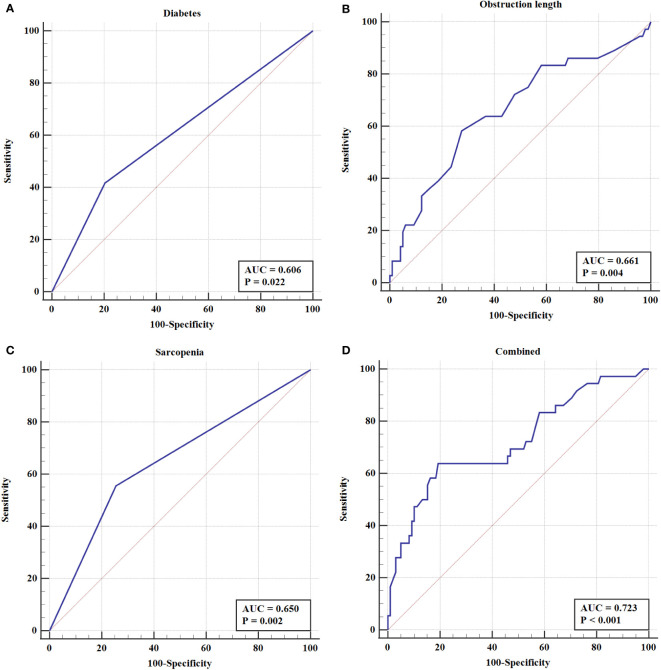
The ROC curve of **(A)** Diabetes **(B)** Obstruction length **(C)** Sarcopenia **(D)** Combined index in EBI after PTBS placement in patients with inoperable BTC.

To further elucidate the predictors on EBI after PTBS placement, the ROC curve was constructed. The results showed that both obstruction length (the areas under the curves, AUC =0.661, *P*=0.004) and sarcopenia (AUC =0.650, *P*=0.002) had better predictive value for EBI than diabetes (AUC=0.606, *P*=0.022). Furthermore, the combined index incorporating obstruction length, sarcopenia and diabetes showed the best predictive value, as the AUC, sensitivity and specificity were 0.723, 63.9%, and 80.6%, respectively (**Table 4** and **Figure 5**).

**Table 4:** ROC analysis of risk factors for EBI in patients after PTBS placement.

Variables	AUC	95% CI	P
Diabetes	0.606	0.518-0.690	0.022
Obstruction length	0.661	0.575-0.741	0.004
Sarcopenia	0.650	0.563-0.731	0.002
Combined	0.723	0.639-0.797	< 0.001

## Discussion

This study is the first to evaluate the association between sarcopenia and EBI after PTBS placement in a retrospective study. The results showed that sarcopenia, obstruction length, and diabetes were independent risk factors of EBI after PTBS placement in patients with unresectable BTC. Considering predictive value, the index combining aforementioned indicators outperformed each one alone. In the present study, the incidence of EBI was 26.9%, including 86% cholangitis, 6% cholecystitis and 8% liver abscess, which was similar to previous reports (15, 16). Zhou et al. (15) developed and validated a nomogram among 243 patients in a multicenter study, where they reported the occurrence of EBI after PTBS was 22.5% in training cohort and 18.0% in validation cohort. Meanwhile, Xu et al. (16) reported that 24% patients developed EBI after PTBS placement. Moreover, bile culture results of bacteria distribution on EBI patients were also comparable to other studies (16). As 47.2% patients had *Escherichia coli*, 16.7% patients had *Enterococcus* and 11.1% patients with unknown results.

Previous studies have explored many risk factors of EBI after PTBS placement, such as diabetes, length and location of obstruction, previous surgical or endoscopic intervention, biliary stones and proton pump inhibitors use (15, 16). However, till now, no studies have explored the effect of sarcopenia on risk of EBI following PTBS placement in BTC patients. In the study, indicators were compared between patients with and without sarcopenia, which unfolded that sarcopenic patients were inclined to show poorer ECOG performance-status and infaust Child-Pugh score. It can be speculated that patients with severely decreased skeletal muscle are commonly ill-nourished and remain poor physiological reserve and resilience, causing poor ECOG performance-status and Child-Pugh score. More importantly, sarcopenic patients were more likely to develop EBI, indicating an association between sarcopenia and EBI. Further analyses confirmed that sarcopenia was a significant risk factor of EBI after PTBS placement in patients with inoperable BTC.

In the present study, sarcopenia was defined based on SMM measured by CT images of the L3 slices, which was considered as one of the gold standards for noninvasive assessment of muscle quantity or mass (10). There is an ongoing debate about the optimal cut-off value for low muscle mass for CT measurements. Prado et al. (19) determined gender-specific cut-off point for SMI to be 52.4 cm^2^/m^2^ for male and 38.5 cm^2^/m^2^ for female patients, which was widely accepted and supported by the international consensus on the definition of cancer cachexia in 2011, however, these cut-offs widely used in western country were determined to best distinguish the difference in survival in a population of obese people with respiratory and gastrointestinal tracts cancer (BMI≥30 kg/m^2^). While Fujiwara et al. (20) indicated that SMI lower than 36.2 cm^2^/m^2^ for men and 29.0 cm^2^/m^2^ for women could be defined as sarcopenia, which could also predict survival in hepatocellular carcinoma patients. According to the Japan Society of Hepatology (JSH) guideline of sarcopenia (21), the definition of sarcopenia was SMI< 42 cm^2^/m^2^ for male and 38 cm^2^/m^2^ for female at L3 vertebra. In our study, sarcopenia was defined based on the lowest tertiles of SMI, and the cut-off point was 42.15 cm^2^/m^2^ and 34.69 cm^2^/m^2^ for male and female, respectively. Considering that skeletal muscle constitution differs in different ethnicity and underlying diseases, novel gender-specific value for SMI in our study could help better risk stratification and perioperative management.

In terms of mechanism, skeletal muscle secretes myokines, which are relatively deficient in sarcopenic patients (22). Many researchers speculated that myokines reduction in sarcopenic patients give rise to immune senescence, fat deposition and insulin resistance, leading to increased proinflammatory factors (23, 24). These processes could be involved in biliary infections after PTBS placement. Previous report supposed that amino acids play a pivotal role in muscle synthesis which can be separated to human organs to generate biological defense responses when body experiences stimulation by some surgical procedure (13). The insufficient amino acids in sarcopenic patients for tissue repair may prolong the time of wound healing and cause further infections (25). In addition, it was said that sarcopenia caused intestinal edema, influencing absorption and metabolism of macronutrients, and ultimately led to malnutrition and infections (26). In the view of the above potential mechanisms, previous studies elucidated that physical exercise combined with supplement of essential amino acid could improve sarcopenia (27), whereby preoperative parenteral nutrition support was shown to be related to a low intra-abdominal infection incidence in sarcopenia patients, which suggested that nutritional support may ameliorate immunosuppression status and boost immune responses to some extent (28). Myostatin inhibitors for targeted treatment of muscle wasting also emerged (29). Further researches are needed to investigate the exact mechanism of these complicated infectious events and assess the association between physical exercise or nutritional support and EBI to improve the management of sarcopenic patients with BTC.

Additionally, obstruction length and diabetes were predictors of EBI after PTBS placement in BTC patients, which were in accord with previous studies. Zhou et al. (15) found that length of obstruction (OR=1.061) and diabetes (OR=5.070) to be independent risk factors of EBI in malignant biliary obstruction patients after PTBS placement, and a calibration plot was constructed, which showed good predictive value. Zhang et al. (30) also reported that biliary stricture length increased risk of deep abdominal infection after biliary intervention. The potential reason may be that long stricture lead to extended operation time, causing bacterial translocation and infection.

There are still controversies about stent position and EBI risk after PTBS placement. Miyayama et al. (31) suggested not covering the cystic duct to avoid cholecystitis when conducting PTBS placement. Huang et al. (32) concluded that bile stent insertion above the main duodenal papilla achieved lower infection rate compared with placement across the main duodenal papilla in a retrospective study. However, Li et al. (6) indicated that stent placement across the main duodenal papilla could help prevent early infectious complications in patients with malignant biliary obstruction. While no significant difference was found between stent position and risk of EBI in our study, there is a trade-off between maintaining sphincter function and stent patency. Therefore, well-controlled randomized studies are essential to better illustrate the relationship between stent position and EBI risk.

There remained some limitations in the study. First, this was a single institutional retrospective study, thus, selection bias cannot be avoided. Second, we did not evaluate other variables related to sarcopenia, including grip strength, chair rise test, and gait speed, owing to its retrospective nature. Third, skeletal muscle was manually delineated to fit the muscle boundaries, and measurement errors were incapable to be completely avoided. Therefore, well-designed prospective study is warranted to reconfirm our results.

## Conclusion

Sarcopenia increased the risk of EBI in patients with inoperable BTC after PTBS placement. Preoperative assessment of sarcopenia may aid in risk stratification and help decision making for clinicians. Determining the relationship between physical exercise or nutritional support and EBI is needed in sarcopenic patients with BTC.

## Data availability statement

The original contributions presented in the study are included in the article/**Supplementary Material**. Further inquiries can be directed to the corresponding author.

## Author contributions

J-HG was involved in the study supervision, study design, and the percutaneous stent placement. QC and XL analyzed and interpreted the data and drafted the manuscript. QC and Z-KW were involved in the critical revision of the manuscript. X-JY, and CF were involved in the data collection and regular follow-up. All authors contributed to the article and approved the submitted version.
