# Supplementary material for: Sarcopenia increases the risk of early biliary infection after percutaneous transhepatic biliary stent placement
Source: Front Oncol. 2022 Dec 9;12:1039987. doi: 10.3389/fonc.2022.1039987 (PMC9780493; doi:10.3389/fonc.2022.1039987)
Supplement: Supplementary file 1 [file Table_1.docx]

Supplementary table 1 Culture results of EBI patients

| Bacteria | Number of patients (%) |
| --- | --- |
| Gram negative |  |
| *Escherichia coli* | 17 (47.2%) |
| *Klebsiella pneumoniae* | 3 (8.3%) |
| *Pseudomonas aeruginosa* | 3 (8.3%) |
| Gram positive |  |
| *Enterococcus* | 6 (16.7%) |
| *Streptococcus* | 2 (5.6%) |
| *Staphylococcus* | 1 (2.8%) |
| Unknown | 4 (11.1%) |
