# Supplementary material for: Sarcopenia increases the risk of early biliary infection after percutaneous transhepatic biliary stent placement
Source: Front Oncol. 2022 Dec 9;12:1039987. doi: 10.3389/fonc.2022.1039987 (PMC9780493; doi:10.3389/fonc.2022.1039987)
Supplement: Supplementary file 2 [file Table_2.docx]

Supplementary table 2 Comparison between patients with or without sarcopenia

| Variable | Sarcopenia | Non-sarcopenia | P |
| --- | --- | --- | --- |
| Total, n (%) | 45 (33.6%) | 89 (66.4%) |  |
| Age, years, median (IQR) | 71 (61-78) | 71 (61-79) | 0.656 |
| Gender, n (%) |  |  | 0.952 |
| Female | 23 (51.1%) | 45(50.6%) |  |
| Male | 22 (48.9%) | 44 (49.4%) |  |
| ECOG, n (%) |  |  | **< 0.001** |
| 0-1 | 15 (33.3%) | 67 (75.3%) |  |
| 2-3 | 30 (66.7%) | 22 (24.7%) |  |
| BMI, kg/m^2^, n (%) |  |  | 0.207 |
| < 25 | 39 (86.7%) | 83 (93.3%) |  |
| ≥25 | 6 (13.3%) | 6 (6.7%) |  |
| Child-Pugh score, n (%) |  |  | **0.002** |
| A | 10 (22.2%) | 44 (49.4%) |  |
| B | 35 (77.8%) | 45 (50.6%) |  |
| Etiology, n (%) |  |  | 0.080 |
| Cholangiocarcinoma | 32 (71.1%) | 70 (78.7%) |  |
| Gallbladder carcinoma | 13 (28.9%) | 14 (15.7%) |  |
| AOV carcinoma | 0 (0%) | 5 (5.6%) |  |
| Obstruction site, n (%) |  |  | 0.377 |
| Low | 9 (20.0%) | 24 (27.0%) |  |
| High | 36 (80.0%) | 65 (73.0%) |  |
| Obstruction length, mm, median (IQR) | 40 (35-44) | 39 (28-43) | 0.320 |
| Diabetes, n (%) |  |  | 0.176 |
| Absence | 30 (66.7%) | 69 (77.5%) |  |
| Presence | 15 (33.3%) | 20 (22.5%) |  |
| Gallstones, n (%) |  |  | 0.506 |
| No | 32 (71.1%) | 68 (76.4%) |  |
| Yes | 13 (28.9%) | 21 (23.6%) |  |
| Pre-procedural PTBD, n (%) |  |  | 0.138 |
| No | 10 (22.2%) | 11 (12.4%) |  |
| Yes | 35 (77.8%) | 78 (87.6%) |  |
| Previous surgical or endoscopic intervention, n (%) |  |  | 0.181 |
| No | 33 (73.3%) | 74 (83.1%) |  |
| Yes | 12 (26.7%) | 15 (16.9%) |  |
| EBI |  |  | **0.001** |
| No | 25 (55.6%) | 73 (82.0%) |  |
| Yes | 20 (44.4%) | 16 (18.0%) |  |
| WBC, 🞨10^9^/L, median (IQR) | 6.6 (4.6-8.4) | 6.7 (5.2-8.3) | 0.724 |
| ALB, g/L, median (IQR) | 33.5 (31.8-36.8) | 34.4 (32.0-37.7) | 0.194 |
| TB, μmol/L, median (IQR) | 118.3  (79.2-186.0) | 81.7 (35.2-176.2) | 0.080 |
| GLU, mmol/L, median (IQR) | 5.6 (4.9-6.5) | 5.1 (4.6-6.4) | 0.118 |

**Abbreviations:** ECOG, Eastern Cooperative Oncology Group; BMI, body mass index; AOV, ampulla of Vater; PTBD, percutaneous transhepatic biliary drainage; EBI, early biliary infection; WBC, white blood cell; TB, total bilirubin; ALB, albumin; GLU, blood glucose.
